# Equitable Participation in Biobanks: The Risks and Benefits of a “Dynamic Consent” Approach

**DOI:** 10.3389/fpubh.2018.00253

**Published:** 2018-09-05

**Authors:** Megan Prictor, Harriet J. A. Teare, Jane Kaye

**Affiliations:** ^1^Melbourne Law School, The University of Melbourne, Carlton, VIC, Australia; ^2^Centre for Health, Law and Emerging Technologies, Nuffield Department of Population Health, University of Oxford, Oxford, United Kingdom

**Keywords:** biobanks, consent, equity, participation, translation, Dynamic Consent, inclusive, communication

## Abstract

Participation in biobanks tends to favor certain groups—white, middle-class, more highly-educated—often to the exclusion of others, such as indigenous people, the socially-disadvantaged and the culturally and linguistically diverse. Barriers to participation, which include age, location, cultural sensitivities around human tissue, and issues of literacy and language, can influence the diversity of samples found in biobanks. This has implications for the generalizability of research findings from biobanks being able to be translated into the clinic. Dynamic Consent, which is a digital decision-support tool, could improve participants' recruitment to, and engagement with, biobanks over time and help to overcome some of the barriers to participation. However, there are also risks that it may deepen the “digital divide” by favoring those with knowledge and access to digital technologies, with the potential to decrease participant engagement in research. When applying a Dynamic Consent approach in biobanking, researchers should give particular attention to adaptations that can improve participant inclusivity, and evaluate the tool empirically, with a focus on equity-relevant outcome measures. This may help biobanks to fulfill their promise of enabling translational research that is relevant to all.

## Introduction: the importance of inclusivity

Participation in health research has tended to be dominated by white, middle-class, higher-educated people in most Western countries, to the exclusion of indigenous groups, the socially-disadvantaged and the culturally and linguistically diverse. This is concerning both from a moral perspective, where equitable access and benefit sharing are valued, and from a scientific perspective that relies on the extrapolation of findings from individual studies to larger population groups to improve health. This bias is also evident in biobank recruitment and affects the wide range of research conducted using biobank samples and data. Biobanks have been used in translational research that seeks to “understand the impact of environmental and other risk exposures on health, understanding genetic risks for common disease, identification of biomarkers in disease progression and prognosis, and are central to the implementation of personalized medicine projects” (1). Therefore, it is important that this bias is recognized and addressed, so that it does not introduce bias in the consideration of new health policy, or perpetuate existing social inequalities. In this paper we will consider whether efforts to achieve representativeness in biobank samples, specifically in terms of the potential for improved recruitment and retention of participants through a “Dynamic Consent” platform (2), can address the known barriers to participation for specific populations.

## Bias in biobanking recruitment

Biobanks, though diverse in size, funding, contents, infrastructure, regulatory and governance frameworks, access policies and purposes, often comprise samples that reflect the lack of diversity and representativeness common to other forms of precision medicine research. Empirical studies have confirmed this, with researchers recently reporting that participants in the UK Biobank—some half a million people aged 40–69 years—were more likely to be female, in better health and living in a less deprived area, than the general population (3). The Estonian Biobank, involving around 5% of the country's population by 2014, reported a bias toward women and younger people compared to the whole population; further, the minority ethnicity Russians in Estonia were underrepresented (4). Similar trends were reported in an analysis of the Dutch Lifelines Study (fewer people were elderly, immigrants, unemployed, had ever smoked, or had less education) (5) and a consortium of Nordic cancer biobanks (cancer rates were less than the general population) (6). While this tendency is not universal [e.g., (7)], the issues of inclusivity and representativeness in large population biobanks are of concern.

Despite the promise of universal benefit that was expected to flow from the Human Genome Project, genetic research has struggled with the same bias toward homogeneity of data sets as other forms of health research—demonstrating a preference for large, “well-characterized, well-powered, predominantly European ancestry cohorts” (8). For example, in genome-wide association studies, researchers reported in 2009 that only around 4% utilized samples with non-European ancestry (9). By 2016 the proportion had risen to almost 20%, but this shift largely reflected the addition of Asian cohorts, with other groups still not represented (10).

While there are many different types of biobanks, cohorts of six or seven figures are becoming increasingly commonplace, as well as associated large-scale longitudinal projects capturing related health and lifestyle data. Biobank managers and funders are increasingly aware of recruitment bias and commonly seek to bolster inclusivity to ensure the representativeness and applicability of their collections. Studies actively seeking to minimize bias include the US-based “All of Us” project, which is “focused especially on enrolling those who have been overlooked in past research studies, and who may represent communities that have inadequately benefited from previous findings” (11). The Gen V project in Victoria, Australia, incorporating newborn heel prick cards, DNA samples and multiple health and education data sets for a 2-year state-wide cohort, is similarly premised on inclusivity (12).

## The effect on research

Diversity of biobank samples is not merely an aspiration for ethical research conduct, it is a scientific imperative. In a paper examining how representative biobanks are of their local populations (7), the authors assert that it is a “primary responsibility [for biobanks] to collect tissues that are a true reflection of their local population and thereby promote translational research, which is applicable to the community.” Oh et al. note that several different factors, including genomic profile and genetic ancestry, inform our comprehension of disease (13). Limiting inclusivity must therefore narrow our understanding of genomics, and stymie progress. Importantly, this selection bias may also be invisible to scientists using the samples–and hence “may be impossible to adjust for in subsequent laboratory or statistical analysis” (14).

If biobanks are not appropriately reflective of the wider population, nor will the research findings derived from them be representative. This raises the risk that research products reaching the clinic will only be relevant for certain sections of the population, which could further exacerbate inequity and health inequalities.

## Recognized barriers to participation

To achieve their recruitment targets, biobank managers and researchers should consider the barriers to inclusion of hard-to-reach groups. This also requires a review of the “[p]olicies for enrolling participants, returning research results and obtaining samples and data [that] can have a far-reaching impact on the type of research that can be performed with each biobank” (1) as well as the research outcomes and results. Several barriers have been identified in the literature that limit the participation in biobanks by all citizens in society.

### Location

Research infrastructures tend to be concentrated in large urban centers, and so distance and transportation (15–17) may have an impact on willingness and capacity to participate in biobank research. However, this is not always the case; the Estonian Biobank has achieved accurate representation from both urban/rural populations (4). Novel solutions such as mobile laboratories might address this problem (18), although these are expensive.

### Literacy and language

People with low literacy, including low health or scientific literacy, or who are linguistically diverse, are sometimes specifically excluded from biobanks. For example, the LifeLines study excluded people who were unable to read Dutch (5). Even absent specific exclusion, they are harder to recruit (16). It may be difficult to find appropriate terminology to explain biobank-relevant concepts in some languages (17, 19). The readability of biobank consent materials can be challenging for the general population (20) and act as a real disincentive to some groups (21) including migrants, those with lower education levels, people with intellectual disability, and children and adolescents.

### Age

There are challenges to the recruitment of both older adults and children to biobanks. For children, these challenges are ethical as well as practical. They are vulnerable subjects with limited but increasing capacity, as they grow older, to understand research and provide truly informed consent (22). Legally, only their parent or legal guardian can consent, but children may provide assent, and have a right to be given information at an appropriate reading level. When they reach adulthood, they can reconsent or withdraw consent for themselves, but re-contacting participants years after the original sample donation can be problematic (23, 24).

Older people are both excluded from, and under-recruited to, healthcare research (25). Age-related health conditions that may affect hearing, vision, cognition, and mobility can impede participation.

### Cultural difference

It is known that “non-White populations are less likely to participate in biobanks and more likely to express concerns about the storage, collection, and use of biospecimens” (26). The considerations driving this reluctance are multifaceted, springing both from cultural values and experiences of exploitation as research subjects.

#### Blood and tissue

Different cultural perspectives around the meaning of blood and tissue samples may weigh heavily on decision-making by indigenous peoples, diverse cultural groups, and minorities around biomedical sample donation (26–28). These include perceptions that samples remain intimately connected with both whole people and groups (29), and hence that the mistreatment of body parts can cause physical or mental harm (30).

#### Individual vs. group decision-making

Some groups have different views as to who can appropriately provide consent, which may diverge from the Western individualist notion of autonomous decision-making. Collective (family or community) engagement, recruitment, and consent has been utilized in settings including some indigenous communities [e.g., Australia, New Zealand, and Alaska (29)], southern Africa (28), and East Asia (31–33). Where research focuses on a specific community, the International Society for Biological and Environmental Repositories endorses community participation in planning for the study and consent processes, specimen use and dissemination of research findings (34). Yet engaging with the appropriate decision-makers in different contexts adds complexity to the biobank recruitment process that can increase costs and time.

#### Mistrust

Indigenous people and minorities have often been subjects of health and anthropological research without consent [with infamous examples including the Tuskegee Syphilis Study (35) and the Havasupai sample research (36)], creating mistrust—and more recently, deep caution on the part of researchers to avoid further harm—that is hard to overcome. In this context, efforts to expand genomic research to address the known lack of ancestral diversity have sometimes backfired. For example, the Human Genome Diversity Project, which arose in the mid-1990s to analyse genetic material to better understand genetic variation, migration and evolution (37), was characterized as the “Vampire Project” by some indigenous groups (38). There remains a clear association between minority status, mistrust, and reluctance to donate biomedical samples (39).

## Online tools to facilitate participation—the example of dynamic consent

Given these challenges, online tools are being developed that might improve engagement, communication and participation with a range of communities. One such tool is Dynamic Consent, which allows research participants to access a digital record of their consent decisions, and to have greater control over how their data and samples are used. Participants can revisit previous decisions and change their mind in real-time via a secure, personal profile. This establishes a channel of communication allowing participants to receive information about the research endeavor, and news and updates as the project progresses. It also enables researchers to manage their communication and engagement strategies with participants and keep a record of interactions.

## Potential benefits of dynamic consent

Dynamic Consent has been discussed as a mechanism that may improve the informed consent process for biobanking (and more widely) by providing research participants with stronger oversight of the uses of their samples and data (2). While Dynamic Consent was originally developed in response to a legal question surrounding consent and decision-making, it has progressed as a mechanism bolstering communication and engagement. Focus groups found that some biobank participants wanted more information about the uses of their samples and data, and the development of the research (40). This emphasis on communication and engagement has led to further discussion around the opportunities offered by a digital patient interface for information delivery, broadening both the range of information provided, and the delivery mode. Several papers reference the potential benefits to be derived from the use of multi-media, and of providing information over time, to counteract the current need to deliver complex information in a single encounter, at the beginning of the research process using a traditional consent approach (2, 41–43).

Given this emphasis on tailored engagement, it is appropriate to examine Dynamic Consent's capacity to enable inclusivity within research endeavors such as biobanks, to overcome some of the barriers outlined earlier. For instance, digital tools and applications have been developed to enable real-time language translation, enabling linguistically diverse communities to contribute on an equal footing. The accessibility of websites is also a significant area of progress; options to work with communities to develop videos, diagrams, and animations have been cited as opportunities for inclusivity and hence diversification of the sample population. This could advance the participation of children by using age-appropriate language and tailored communication techniques, as well as enhancing accessibility to indigenous communities. The animation developed to explain the historical samples held in the National Centre for Indigenous Genomics is an excellent example (44).

Alongside these specific tools for accessibility is the philosophy behind Dynamic Consent which emphasizes the importance of timing. This includes: the timing of recruitment, and allowing participants to reconsider their decisions at home; the timing of information provision, such that it can be cascaded at different points throughout the research endeavor; and recognition that biobanking often takes place over years, if not decades. Having a system that allows participants to check in at their own convenience, over time, and be reminded of their involvement in research, is itself inclusive. It can help to facilitate the informed decision-making of adults who contributed to biobanks as children about their continued participation, and might mean that fewer are lost to follow-up (24). This ongoing connection might also foster trust by promoting improved understanding of the biobank and its research outputs, and a stronger sense of collaboration between donors and researchers. It could thus help to overcome cultural concerns about the treatment of blood and tissue samples, through promoting greater transparency and accountability.

## Potential drawbacks and the need for further evaluation

Notwithstanding these significant benefits, there are also concerns about the challenges such technology will create. The most obvious is its potential to exacerbate exclusion and disenfranchisement, if participants are not willing to engage using the technology. This is often cited as a “digital divide,” with focus (perhaps unfairly) on older generations. However, it could equally apply to communities, for instance in remote parts of Australia, that lack access to technology, or reliable infrastructure including Wifi networks to allow meaningful reliance on these tools (45, 46). Some research on the willingness of different populations to engage with health information electronically has had encouraging findings (47, 48). However, this needs to be examined more carefully and tested with a diverse range of people, before researchers can rely on solely on digital tools. The move toward whole population electronic health records, such as the My Health Record in Australia (49), offers an opportunity to apply empirical methods, including focus groups and qualitative interviews, to investigate the willingness and capacity of different groups to engage with digital health tools.

While several projects already use Dynamic Consent [e.g., RUDY (50), CHRIS (42), PEER (51)], further evaluation is needed to gather real-world data on its influence on the participant experience, people's understanding of the research endeavor, and their sense of oversight and control. Within Dynamic Consent, consent choices are carefully set out to allow granular decision-making and tailored involvement. This approach should mitigate the challenges commonly encountered with online terms and conditions for products and services, including software and applications, that discourage full consideration of long legal explanations, and instead allows an easy and informative tick-box agreement (52). However, this will need to be explored as the projects using Dynamic Consent reach maturity, and it becomes possible to examine its influence on consenting behavior. Any risk that Dynamic Consent might further discourage participants from carefully considering important aspects of the research, or might promote “consent fatigue,” would undermine the intentions of the approach. Dynamic Consent interfaces must be designed to support people to think through important aspects of biobank sample donation, while not overwhelming them or causing further disengagement. Solutions may be found in citizen-science programmes using gamification to maintain interest, for example the project Foldit (53), which encourages members of the public to help describe the structures of proteins (54).

Accessibility challenges have and can be addressed using information technology. Examples include providing larger text or audio tools for visually-impaired people, and deploying a keyboard or joystick for those with certain physical impairments. Nonetheless, there is still concern that digital consenting tools will not be appropriately tailorable for all needs, and that biobanks will lack the resources to finance the variety of tools necessary to support accessibility. The needs of specific populations must be carefully considered for each project, to balance what is needed against what is practical, and to enhance inclusivity. This will not only relate to the consenting tools, but to the wider requirements of the biobank, and will rely upon an ongoing conversation with the target population.

Researchers conducting medical research, including using biobanks, are increasingly concerned about managing results that could be of immediate significance for a participant's health, and may also be relevant for their family or wider community. This raises a question about how to support consent relating to communication of findings, for the participant, but also whether (and if so how) family or community consent needs to be developed (31). Within genomics and precision medicine, projects involving multiple family members, often over several generations, are vital in building a clearer picture of different diseases and conditions (55). These projects rely on agreement from all family members, and could be undermined if one person was to withdraw. This raises questions for diversity and inclusion, as it may lead to family groups with particular characteristics being supportive of research, and may exacerbate some of the issues experienced with individual participation. Dynamic Consent has not yet been developed to support “family consent,” however it should be possible to allow records to be linked and unlinked based on each family member's decisions.

## Next steps

Considering the implications of narrow participation for the generalizability of translational research arising from biobanks, further attention must be given to mechanisms that could overcome barriers to inclusivity. Dynamic Consent holds the potential to improve the engagement of different groups through its flexibility and adaptability, and its focus on participant empowerment over an extended period. Further to the examples of Dynamic Consent mentioned above, it will be important for biobank managers and researchers to experiment with applications of the approach that focus on addressing barriers and facilitating widespread involvement. These applications could, for instance, incorporate child-friendly language, multiple translations, and culturally-tailored explanations. The relevance of biobank-based research to family members and communities should be reflected in tools that can accommodate decision-making by groups as well as individuals, and multi-generational sample donation.

There is also a need for empirical research on the effects of Dynamic Consent tools on equity-oriented research participation outcomes. The approach should be tested in high-quality evaluations reporting on outcome measures that are able to show if there is a difference between more and less advantaged groups (56, 57). The PROGRESS-Plus characteristics (58, 59) provide a sound basis for the design of Dynamic Consent research applying an equity lens. Attending to these concerns will help to ensure that biobanks fulfill their promise as a driver of translational research having benefits for all patients, not just some. Dynamic Consent cannot overcome all challenges to inclusivity; the starting point is for researchers to consider which populations need better representation, and to work alongside communities to design methods for inclusion. Once recruitment strategies have been devised, however, Dynamic Consent can support participants, and ensure a partnership approach to research that might motivate future involvement, and for participants to encourage friends and relatives to participate too.

## Author contributions

MP and HT planned the manuscript with input from JK. All authors compiled the first draft of the manuscript. MP and HT revised the manuscript. MP finalized and submitted the manuscript after all authors had critically read, revised, and approved the final version.

### Conflict of interest statement

The authors declare that the research was conducted in the absence of any commercial or financial relationships that could be construed as a potential conflict of interest.
